# Microencapsulation Technology: A Powerful Tool for Integrating Expansion and Cryopreservation of Human Embryonic Stem Cells

**DOI:** 10.1371/journal.pone.0023212

**Published:** 2011-08-05

**Authors:** Margarida Serra, Cláudia Correia, Rita Malpique, Catarina Brito, Janne Jensen, Petter Bjorquist, Manuel J. T. Carrondo, Paula M. Alves

**Affiliations:** 1 Instituto de Tecnologia Química e Biológica, Universidade Nova de Lisboa, Oeiras, Portugal; 2 Instituto de Biologia Experimental e Tecnológica, Oeiras, Portugal; 3 Cellartis AB, Gotemborg, Sweden; 4 Faculdade de Ciências e Tecnologia, Universidade Nova de Lisboa, Monte da Caparica, Portugal; Michigan State University, United States of America

## Abstract

The successful implementation of human embryonic stem cells (hESCs)-based technologies requires the production of relevant numbers of well-characterized cells and their efficient long-term storage. In this study, cells were microencapsulated in alginate to develop an integrated bioprocess for expansion and cryopreservation of pluripotent hESCs. Different three-dimensional (3D) culture strategies were evaluated and compared, specifically, microencapsulation of hESCs as: i) single cells, ii) aggregates and iii) immobilized on microcarriers. In order to establish a scalable bioprocess, hESC-microcapsules were cultured in stirred tank bioreactors.

The combination of microencapsulation and microcarrier technology resulted in a highly efficient protocol for the production and storage of pluripotent hESCs. This strategy ensured high expansion ratios (an approximately twenty-fold increase in cell concentration) and high cell recovery yields (>70%) after cryopreservation. When compared with non-encapsulated cells, cell survival post-thawing demonstrated a three-fold improvement without compromising hESC characteristics.

Microencapsulation also improved the culture of hESC aggregates by protecting cells from hydrodynamic shear stress, controlling aggregate size and maintaining cell pluripotency for two weeks.

This work establishes that microencapsulation technology may prove a powerful tool for integrating the expansion and cryopreservation of pluripotent hESCs. The 3D culture strategy developed herein represents a significant breakthrough towards the implementation of hESCs in clinical and industrial applications.

## Introduction

Human pluripotent stem cells, including embryonic stem cells (hESCs) and induced pluripotent stem cells (hiPSCs) constitute an exciting emerging field. The inherent capacity of these cells to grow indefinitely (self-renewal) and to differentiate into any mature cell of the human body (pluripotency) makes them extremely attractive for regenerative medicine, tissue engineering, drug discovery and toxicology [1]. However, the establishment of effective and robust protocols for large-scale expansion, storage and distribution of hESCs is imperative for the development of high quality therapeutic products and/or functional screening tools.

hESCs are routinely cultured in two-dimensional (2D) systems, namely Petri dishes, well-plates and tissue culture flasks [2]. In recent years, the inadequacy of conventional 2D culture systems in mimicking the *in vivo* microenvironments of stem cell niches has proven a constant shortcoming in both basic biology and tissue engineering studies [3]. Despite the importance of cell-cell and cell-matrix interactions in hESC cultivation, they have not yet been properly addressed in these systems. In addition, the inherent variability, lack of environmental control and low production yields associated with these culturing approaches are the main drawbacks hampering the development of efficient, scalable and cost-effective stem cell expansion processes (reviewed in [4]). The low cell recovery yields and the high rates of uncontrolled differentiation obtained after cryopreservation [5] also limit the use of the 2-D systems in clinical and/or industrial applications.

Much effort has been invested in the development of more efficient hESC culture systems, namely by combining a strategy for 3D cell organization with a bioreactor-based system where scalability, straightforward operation and homogeneous culture environment are guaranteed [6], [7]. Recent studies have shown the successful use of stirred tank bioreactors (spinner vessels and environmentally controlled stirred tank bioreactors) for expanding hESCs as aggregates or immobilizing them on microcarriers [6], [7], [8]. From a clinical/industrial perspective, these systems still require further improvements in order to increase cell expansion yields and ensure efficient bioprocess integration with cryopreservation protocols. In fact, stirred culture vessels often apply mechanical forces (mixing and occasionally perfusion) to the cells, which may ultimately compromise cell viability, morphology, gene expression and differentiation potential [9]. The excessive aggregate/microcarrier clumping observed during culture is another concern since it may lead to the formation of necrotic centers and/or promote spontaneous differentiation, reducing cell expansion yields. Moreover, the development of effective cryopreservation protocols capable of ensuring efficient cell storage and transportation after large-scale expansion is still lacking. Although Nie *et al* reported a new method for the cryopreservation of hESCs adherent on microcarriers [10], this protocol needs further optimization in order to remove animal feeder cells and improve cell attachment/survival after thawing.

Cell microencapsulation technology is an attractive approach for overcoming the bioprocess challenges mentioned above since it provides cell protection from hydrodynamic shear and prevents excessive aggregate agglomeration while allowing for the efficient diffusion of nutrients, growth factors and gases through the microcapsule matrix [11]. Several hydrogels have been used in hESC culture including alginate [12], poly(lactic-co-glycolic acid)/poly(l-lactic acid) scaffolds [13], agarose [14], chitosan [15] and hyaluronic acid [16]. Alginate is the most common encapsulation material due to its intrinsic properties including biocompatibility, biosafety and permeability [17]. The production of alginate cell-microcapsules can be performed under safe and physiological conditions (e.g. physiological temperature and pH, use of isotonic solutions instead of cytotoxic solvents) [18] and using good manufacturing practice (GMP) guidelines [19], a fact which potentiates the use of this technology in cell-based therapies. Indeed, the great potential of alginate microcapsules for transplantation of Langerhans' islets and other factor-secreting cells and tissues has already been reported [20], [21].

Cell microencapsulation in alginate has been adopted by our group and others to improve the viability and functionality of primary hepatocytes [22], [23] and to enhance the differentiation of stem/progenitor cells into different cell types in bioreactors [24], [25], [26], [27], [28]. In addition, we recently demonstrated that cell encapsulation in alginate is a valuable strategy for improving cell viability and the integrity of cell monolayers and neurospheres after freeze/thawing, since cells are protected against mechanical damages during ice crystallization and the risk of disrupting cell-cell and cell-matrix contacts are reduced through immobilization within the hydrogel [29], [30]. Despite such success in many (stem) cell types, studies describing the microencapsulation of hESCs are still limited [12], [25], [31].

This paper reports the first efficient integrated bioprocess for the expansion and cryopreservation of hESCs using cell microencapsulation in alginate. Different strategies were evaluated and compared including microencapsulation of i) single cells, ii) cell aggregates and iii) cells immobilized on microcarriers, since each approach allows for different cell-cell/matrix interactions. Microcapsules containing hESCs were cultured in stirred tank bioreactors (spinner vessels) and, after expansion, cryopreserved in cryovials, in order to develop a scalable and straightforward bioprocess.

## Materials and Methods

### hESCs culture on feeder layer

hESCs (SCED™461, Cellartis AB, Göteborg, Sweden) were routinely propagated as colonies in static systems (6 well-plates) on a feeder layer of human foreskin fibroblasts (hFF, ATCC collection), inactivated with mitomycin C (Sigma-Aldrich, Steinheim, Germany), in DMEM-KO culture medium (Knockout™-DMEM supplemented with 20% (v/v) Knockout-Serum Replacement (KO-SR), 1% (v/v) MEM non-essential amino acids (MEM-NEAA), 0.1 mM 2-mercaptoethanol, 2 mM Glutamax, 1% (v/v) Pen/Strep, 0.5% (v/v) Gentamycin (all from Invitrogen, Paisley, UK)) and 10 ng/mL basic fibroblast growth factor bFGF (Neuilly-Sur-Seine, France, Peprotech), as previously described [2]. Every 10–12 days, i.e. when hESC colonies covered approximately 75–85% of the surface area of the culture well, they were digested with TrypLE™ Select (Invitrogen, Paisley, UK) for 6–8 minutes, and the single cell suspension was transferred to freshly inactivated hFF feeders (at splitting ratios between 1∶4 and 1∶24). The culture medium was replaced every 1–3 days.

### Preparation of mEFs conditioned medium

For the production of conditioned medium (mEF-CM), mouse embryonic fibroblasts (mEFs, Millipore, Billerica, MA, USA) were mitotically inactivated and replated on gelatin-coated T-flasks (Nunc, Roskilde, Denmark) at 5.5×10^4^ cell/cm^2^ in DMEM-KO medium without bFGF (0.5 mL/cm^2^). Briefly, inactivated mEFs were cultured at 37°C with 5% (v/v) CO_2_ (in air) and conditioned media were collected daily for a total of 10 days per batch. Before feeding to hESC cultures, mEF-CM was filtered and supplemented with 10 ng/mL bFGF and 0.1 nM Rapamycin (Sigma, Steinheim, Germany).

### Microencapsulation of hESCs

#### Alginate

Ultra Pure MVG alginate (UP MVG NovaMatrix, Pronova Biomedical, Oslo, Norway) was prepared at a concentration of 1.1% (w/v) in 0.9% (w/v) NaCl solution [31].

#### Microcapsule formation

Microcapsules were prepared by passing the alginate-cell mixture using a 1 mL syringe through an air-jet generator as described elsewhere [22], [23], [32] at an air flow rate of 2–3.5 L/min and an air pressure of 1 bar. These encapsulation conditions yielded microcapsules with a diameter of approximately 500–700 µm. For cross-linkage of the UP MVG alginate, a 100 mM CaCl_2_/10 mM HEPES solution adjusted to pH 7.4 was used. Alginate microcapsules were washed twice with 0.9% (w/v) NaCl solution and once with DMEM-KO medium before being transferred to culture systems.

#### Alginate microcapsules dissolution

Ca^2+^-UP MVG alginate was dissolved by incubating the microcapsules with a chelating solution (50 mM EDTA and 10 mM HEPES in PBS) for 5 min at 37°C [31].Cells were washed twice with PBS and incubated with culture medium until further analysis.

### Three-dimensional (3D) hESC cultures

**Figure 1:**
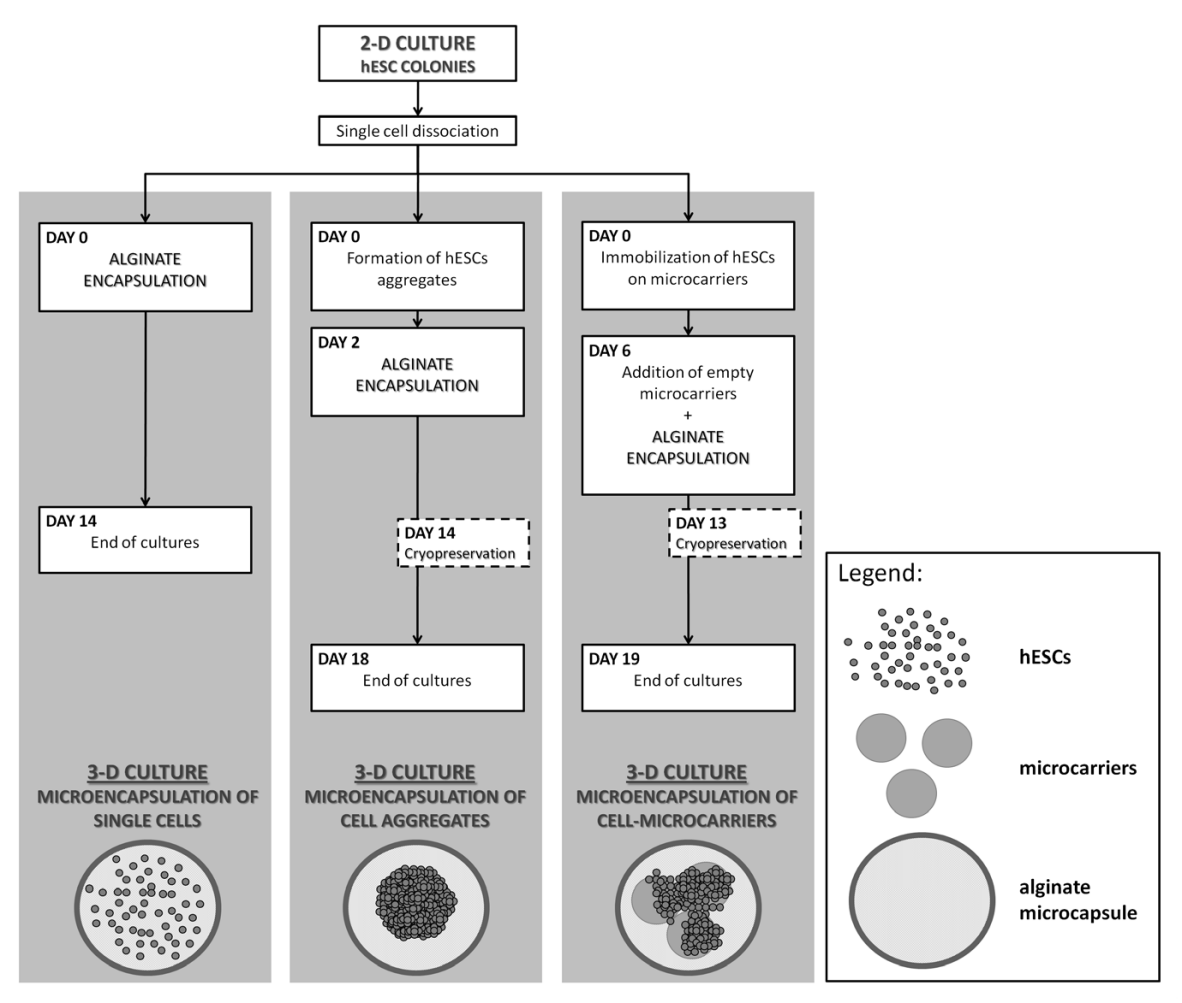
Main steps of the microencapsulated 3D culture strategies developed for expansion and cryopreservation of hESCs.

Figure 1 describes the main steps of the 3D culture strategies developed.

#### Encapsulation of single cells

Before detachment from 2D static cultures, hESCs colonies were pre-treated for 1 h with 5 µM Y-27632, a selective Rho kinase (ROCK) inhibitor (ROCKi, Calbiochem Nottingham, UK). The single cell suspension, obtained after dissociating the colonies with TrypLE Select, was immediately encapsulated at different concentrations in alginate (0.75, 2 and 3×10^6^ cell/mL alginate). hESCs-microcapsules were then inoculated into 125 mL Erlenmeyer (Corning, Corning, NY, USA) and cultured in 15 mL mEF-CM supplemented with 10 µM ROCKi, at 37°C and 5% CO_2_ in an orbital shaker with an agitation of 70 rpm. In all conditions tested, cells were inoculated at 1.5×10^5^ cell/mL.

#### Encapsulation of hESC aggregates

hESCs were dissociated from the 2D static cultures and inoculated as single cells at 1.5×10^5^ cell/mL into Erlenmeyer (Corning, Corning, NY, USA). Cells were cultured in 50 mL mEF-CM supplemented with 10 µM ROCKi, at 37°C and 5% CO_2_, using an orbital agitation of 70 rpm. Encapsulation was performed at day 2; aggregates were pre-treated with 5 µM ROCKi for 1 h and then transferred to 15 mL tubes to allow them to deposit and be removed from the culture medium. After the addition of alginate, aggregates were encapsulated, transferred to 125 mL spinner vessels (Wheaton, Techne, NJ, USA) equipped with paddle impellers and cultured in 100 mL of mEF-CM at 45 rpm for an additional 16 days. The culture medium was partially replaced three times a week by stopping agitation (to induce microcapsule deposition), removing 50% of the medium and feeding with 50% of fresh medium. Cultures of non-encapsulated aggregates were also performed in parallel and used for control purposes. Both cultures were monitored for cell viability, metabolic activity, aggregate size, concentration and composition throughout the experiments. For flow cytometry analysis, aggregates were transferred to gelatin coated surfaces, in mEF-CM, where cells were able to migrate. After 2–3 days, cells were dissociated using TrypLE Select and processed for flow cytometry analysis using the protocol described below.

#### Encapsulation of hESCs immobilized on microcarriers

hESCs were inoculated at 4.5×10^5^ cell/mL into 125 mL spinner vessels with paddle impellers containing Cytodex3™microcarriers (2 g/L, GE Healthcare, Uppsala, Sweden). The microcarriers were prepared and sterilized according to the manufacture's recommendation and coated with Matrigel (BD Biosciences, Bedford, MS, USA) as described in the literature [7]. Cells were cultured in 25 mL of mEF-CM supplemented with 10 µM ROCKi, and the spinner vessels were placed inside an incubator at 37°C, 5% CO_2_ under intermittent stirring. After 6 h, fresh mEF-CM was added to the cultures and the agitation rate was set to 24 rpm. By day 3, more media was added for a final volume of 100 mL. The encapsulation was performed at day 6; empty microcarriers (1 or 2 g/L) coated with Matrigel were added to the cultures 1 h before encapsulation. During this period, cultures were treated with 5 µM ROCKi. After encapsulation, hESCs were transferred to spinner vessels and cultured in the same conditions for an additional 13 days. Fifty percent of the medium was replaced daily. Cultures of non-encapsulated cells-microcarriers were also performed and run in parallel as a control. Both cultures were monitored for cell concentration, viability and culture composition over time.

At the end of the expansion process of both cell aggregates and hESC-microcarrier cultures, microcapsules were dissolved,using the protocol described above (section- microencapsulation of hESC),and hESC clumps were dissociated and plated on top of a monolayer of inactivated hFF to further assess cell pluripotency.

### Cell cryopreservation

Cultures of non-encapsulated and encapsulated hESCs were harvested from the spinner vessels and cryopreserved using the slow freezing rate method [30]. The hESC-microcarriers and hESC-aggregates were collected at day 13 and 14 of culture, respectively (Figure 1), and all samples were pre-treated with 5 µM ROCKi for 1 hour before cryopreservation.

#### Freezing

At freezing, after the deposition of the microcapsules, the culture medium was removed and the cryopreservation medium (90% KO-SR, 10% (v/v) DMSO (Sigma, Steinheim, Germany), 5 µM ROCKi) was added. Cell suspensions were then transferred to cryovials (Nunc, Roskilde, Denmark) (1 mL/vial). The cells were allowed to equilibrate in the cryopreservation medium for 20 minutes at 4°C. Samples were frozen to −80°C in an isopropanol-based freezing system, (“Mr. Frosty”, Nalgene, NY, USA) at a rate of 1°C per minute, and stored in the gas phase of a liquid nitrogen reservoir until thawing.

#### Thawing

Following storage, cells were quickly thawed by placing the cryovials in a 37°C water bath; a stepwise dilution (1∶1, 1∶2, 1∶4) in mEF-CM was performed immediately afterwards in order to dilute the DMSO while reducing osmotic shock [30]. Cells-microcapsules were transferred to Petri-dishes and cultured for 9 days in mEF-CM supplemented with 5 µM of ROCKi. Media exchange was performed daily. At day 9, microcapsules were dissolved and hESC clumps were dissociated with TrypLE Select; hESCs were transferred to a monolayer of inactivated hFF and maintained in culture for several passages for post-thaw studies of growth and pluripotency.

#### Assessment of hESC survival after thawing

The percentage of hESCs survival/recovery after thawing was determined by calculating the ratio between the number of viable hESCs after cryopreservation and the number of initially frozen viable hESCs, counted using a Fuchs-Rosenthal haemocytometer chamber (Brand, Wertheim, Germany) and the Trypan Blue (Invitrogen, Paisley, UK) exclusion method.

### Evaluation of cell viability

Three methods were used to estimate cell viability.

#### Cell membrane integrity assay

The qualitative assessment of the cell plasma membrane integrity during culture was performed using the enzyme substrate fluorescein diacetate (FDA; Sigma-Aldrich, Steinheim, Germany) and the DNA-dye propidium iodide (PI; Sigma-Aldrich, Steinheim, Germany) as described in the literature [7]. Briefly, cells/microcapsules were incubated with 20 µg/mL FDA and 10 µg/mL PI in PBS for 2–5 min and then observed using fluorescence microscopy (Leica Microsystems GmbH, Wetzlar, Germany).

#### Trypan Blue exclusion method

The total number of viable cells was determined by counting the colorless cells in a Fuchs-Rosenthal haemocytometer chamber after incubation with Trypan Blue dye (0.1% (v/v) in PBS).

#### Lactate dehydrogenase (LDH) activity

LDH activity from the culture supernatant was determined by monitoring the rate of oxidation of NADH to NAD^+^ coupled with the reduction of pyruvate to lactate at 340 nm. The specific rate of LDH release (q_LDH_) was calculated for each time interval using the following equation: qLDH = (ΔLDH)/( Δt×ΔXv), where ΔLDH is the change in LDH activity over the time period Δt, and ΔXv is the average of the total cell number during the same period. The cumulative value q_LDHcum_ was estimated by q_LDHcum i+1_ = q_LDHi_+q_LDH i+1_.

### Evaluation of metabolic activity

#### AlamarBlue™ assay

hESCs metabolic activity was assessed using the metabolic indicator alamarBlue according to the manufacture's recommendation (Paisley, UK, Invitrogen). Briefly, 2 mL of hESC culture were incubated overnight with fresh medium containing 10% (v/v) alamarBlue. Fluorescence was measured in 96-well plates using a microwell plate fluorescence reader (FluoroMax-4, Horiba JobinYvon).

### Evaluation of cell growth

#### Apparent growth rate (μ_app_)

μ_app_ was estimated using a simple first-order kinetic model for cell expansion: dX/dt = μX, where t (day) is the culture time and X (cell) is the value of viable cells for a specific t. The value of μ was estimated using this model applied to the slope of the curves during the exponential phase.

#### Expansion ratio or fold increase (FI) in cell concentration

FI was evaluated based on the ratio X_MAX_/X_0_, where X_MAX_ is the peak of cell density (cell/mL) and X_0_ is the lowest cell density (cell/mL).

### Characterization of hESCs

For all culture samples, microcapsules were dissolved prior to analysis using the protocol described above (section- microencapsulation of hESC). The undifferentiated status of hESCs was evaluated by analyzing the activity of alkaline phosphatase (AP) and by detecting the expression of specific stem cell and pluripotency markers using immunocytochemistry and flow cytometry analysis.

#### Alkaline Phosphatase (AP) staining

Cultures were stained using an AP activity detection kit (Millipore, Billerica, MA, USA) according to the manufacturer's instructions and observed using an inverted phase contrast microscope (Leica Microsystems GmbH).

#### Immunocytochemistry

Cultures of hESC were fixed in 4% (w/v) paraformaldehyde (PFA) in PBS for 20 minutes, permeabilized (only for detection of intracellular markers Oct-4 and Nanog) for 5 minutes in 0.1% (w/v) Triton X-100 (Sigma-Aldrich, Steinheim, Germany) in PBS and subsequently incubated with primary antibody overnight at 4°C. Cells were washed three times in PBS and then incubated with secondary antibodies during 1 h at room temperature in the dark. After three washing steps with PBS, cell nuclei were counterstained with 4,6-diamidino-2-phenylindole (DAPI, Sigma-Aldrich, Steinheim, Germany). Cells were visualized using spinning disk confocal (Nikon Eclipse Ti-E, confocal scanner: Yokogawa CSU-x1) and inverted (Leica Microsystems GmbH) fluorescence microscopy. In samples of hESC aggregates, an additional permeabilization step was performed before the addition of primary antibodies; cells were incubated with 0.2% fish skin gelatin and 0.1% TX-100 in PBS for 2 h at room temperature. Primary antibodies used were: Tra-1-60, Tra-1-81, Oct-4 (all from Santa Cruz Biotechnology, Santa Cruz, CA, USA) and Nanog (Millipore). Secondary antibodies used were: goat anti-mouse IgM-AlexaFluor488 and goat anti-mouse IgG-AlexaFluor 488 (all from Invitrogen, Paisley, UK).

#### Flow cytometry

Cell clumps were dissociated with TrypLE Select and the single cell suspension was re-suspended in washing buffer (WB) solution (5% (v/v) FBS in PBS). After two washing steps, cells were incubated with primary antibody for 1 h at 4°C, washed three times in WB and then incubated with the secondary antibody for additional 30 min at 4°C. After 2 washing steps with WB, cells were analyzed in a CyFlow® space (PartecGmbH, Münster, Germany) instrument as reported elsewhere [16]. Ten thousand events were registered *per* sample. Primary antibodies used were: Tra-1-60, SSEA-4, SSEA-1 and isotype control antibodies (all Santa Cruz Biotechnology, Santa Cruz, CA, USA) and hES-Cellect™ (Cellartis AB, Göteborg, Sweden). Secondary antibodies used were: goat anti-mouse IgM-AlexaFluor488 and goat anti-mouse IgG-AlexaFluor 488 (all from Invitrogen, Paisley, UK).

### In vitro pluripotency

The cell pluripotency was evaluated *in vitro* via embryoid body (EB) formation and spontaneous differentiation. Microcapsules were dissolved as described above and hESCs dissociated, transferred to non-adherent Petri dishes (5×10^5^ cell/mL) and cultured in suspension for 1 week in DMEM-KO medium without bFGF. EBs formed during this time were harvested and cultured in gelatin-coated plates for a further 2 weeks (the medium was changed three times a week). Differentiated cells were identified using immunocytochemistry as described above. Primary antibodies used were: α-smooth muscle actin (DAKO, Glostrup, Denmark), Forkhead box A2 (Santa Cruz Biotechnology, Santa Cruz, CA, USA) and β tubulin type III (Chemicon, Temecula, CA, USA). Secondary antibodies used were: goat anti-mouse IgG-AlexaFluor488 and donkey anti-goat IgG-AlexaFluor594 (all from Invitrogen, Paisley, UK).

### Data analysis and statistics

All data presented show n>2 replicates. Error bars denote the standard deviation of the mean. For membrane integrity and immunofluorescence microscopy, representative photographs are depicted.

Statistical difference between encapsulated and non-encapsulated cultures was assessed using single factor ANOVA. A 95% confidence level was considered to be statistically significant.

## Results

Results previously reported by our group and others demonstrate that it is possible to expand hESCs as aggregates or when immobilized on microcarriers in stirred tank bioreactors [6], [7], [8]. Attempting to further increase the cell expansion yields, different 3D cell microencapsulation strategies were evaluated. The most promising strategies were selected to assess the impact of microencapsulation on cell cryopreservation, with the goal of implementing an integrated bioprocess for the robust expansion and storage of pluripotent hESCs. In this work, calcium 1.1% (w/v) UP MVG alginate microcapsules were used since the properties of this matrix fulfill the main requisites (permeability, stability and elasticity) for supporting an efficient hESC culture [31].

### Expansion of microencapsulated hESCs as single cells

We first investigated the hypothesis of expanding single hESCs in alginate microcapsules. Cells were encapsulated at different concentrations, 0.75, 2 and 3×10^6^ cell/mL alginate, and inoculated at 1.5×10^5^ cell/mL in stirred culture systems. For all conditions tested, cell viability decreased gradually from approximately 95% to 5% after 7 days of cultivation (Figure S1). When a higher cell concentration was used (3×10^6^ cell/mL alginate), viable cell aggregates were observed in culture from day 7 onwards, indicating that some cells remained viable and proliferated. However the percentage of populated microcapsules was very low (<10%, data not shown). These results indicate that the microencapsulation of single cells is not a suitable strategy for expanding hESCs.

### Expansion of microencapsulated hESC aggregates in stirred tank bioreactors

For the second strategy, hESCs were induced to form small cell aggregates after single cell enzymatic dissociation (Figure 1). By day 2, aggregates ranging from 30–100 µm were encapsulated to generate approximately 1 aggregate per microcapsule, and transferred to spinner vessels.

**Figure 2:**
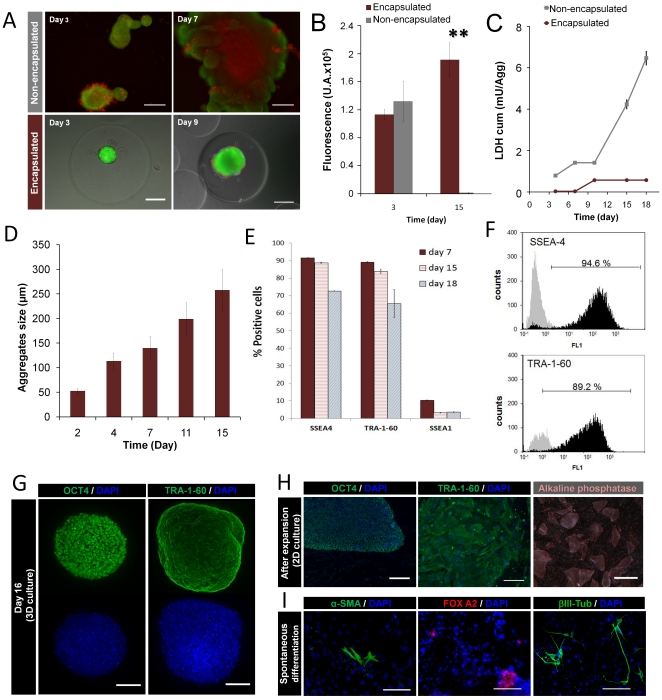
Effect of alginate microencapsulation on the expansion of hESC as aggregates. hESC aggregates were encapsulated at day 2 and cultured in spinner vessels. (**A**) Phase contrast and fluorescence images of encapsulated and non-encapsulated cultures at days 3, 7 and 9. Viability of hESC aggregates assessed by staining with fluoresceine diacetate (FDA-live cells, green) and propidium iodide (PI- dead cells, red). Scale bar: 100 µm. (**B–C**) Cell growth performance of both encapsulated (purple) and non-encapsulated (grey) cultures. (**B**) Metabolic activity measured by alamarBlue test on the day after microencapsulation (day 3) and at day 15. Error bars denote SD of 3 measurements. ** indicates significant difference (P<0.05) in metabolic activity by one-way ANOVA analysis. (**C**) Cumulative values of specific rates of LDH release overtime. Error bars denote SD of 3 measurements. (**D**) Aggregate size of encapsulated cultures at days 2, 4, 7 and 15 of culture. Error bars denote SD of 10 measurements. (**E–I**) Characterization of encapsulated hESC aggregates expanded in spinner vessels. (**E**) Percentage of SSEA-4, TRA-1-60 and SSEA-1 positive cells at days 7 (purple bars), 14 (pink stripes bars) and 21 (grey stripes bars). Error bars represent SD of 2 measurements. (**F**) Flow cytometry analysis of SSEA-4 and TRA-1-60 positive cells at day 7 of culture. (**G**) Confocal images of aggregates labeled for Oct-4 and TRA-1-60 at day 16 of 3D culture. Scale bar: 50 µm. (**F–G**) Flow cytometry analysis of the expanded population (**H**) Immunofluorescence images of Oct-4 and TRA-1-60 labeling and phase contrast pictures of alkaline phosphatase (AP) activity, staining after expansion (2D culture). Nuclei were labeled with DAPI (blue). Scale bars: immunofluorescence images - 200 µm, AP image −1 mm. (**I**) *In vitro* pluripotency analysis. Microcapsules were dissolved and hESCs were transferred to a monolayer of inactivated hFF. At confluence, colonies were dissociated and hESCs were able to form embryoid bodies (EBs) in non-adherent conditions and differentiated into cells from all three germ layers. Fluorescence images of differentiated cultures labeled for α–SMA (α smooth muscle actin, mesoderm), FOX-A2 (Forkheadbox A2, endoderm) and βIII-Tub (β tubulin type III, ectoderm). Nuclei were stained with DAPI (blue). Scale bar: 100 µm.

The results show that the microencapsulation of aggregates enhanced the culture performance of hESCs as compared to the microencapsulation of single cells. Aggregates of hESC presented high cell viability and a spherical shape during culture time (Figure 2A). After 2 weeks, an increase in aggregate size (5-fold, Table 1, Figure 2D) and in metabolic activity (2-fold, Figure 2B and Table 1) was observed, indicating that hESCs proliferated inside alginate microcapsules. Overall, a significant improvement in cell viability and metabolic activity was obtained as compared to non-encapsulated cultures (P<0.05) where aggregates clumped together and formed large (>1 mm in size) irregular structures with necrotic centers (Figure 2A). In fact, the pronounced decrease in metabolic activity and the high values of cumulative LDH release confirm that the culture of non-encapsulated hESC aggregates in spinner vessels resulted in substantial cell death (Figure 2B, C).

**Table 1:** Expansion and cryopreservation of encapsulated and non-encapsulated hESC cultures.

Culture Strategy	hESC aggregates	
Alginate Microencapsulation	No	Yes
**EXPANSION**		
**Fold increase in metabolic activity** (2weeks)	0	2.4±0.2
**Initial aggregate size** (day 2) (µm)	53±16	53±16
**Final aggregate size** (day 15) (µm)	-	257±61
**CRYOPRESERVATION**		
**% cell survival**	0%	0%

Culture Strategy	hESCs immobilized on microcarriers		
Alginate Microencapsulation	No	Yes	Yes
Microcarrier Concentration	3 g/L	3 g/L	4 g/L
**EXPANSION**			
**Initial cell concentration**(×10^5^ cell/mL)	1.7±0.3	1.8±0.1	1.5±0.6
**Maximum cell concentration**(×10^5^ cell/mL)	12.7±0.5	19.0±2.4	28.2±3.8
**Expansion ratio/Fold increase related to initial cell concentration**	7.7±0.2	10.7±0.8	19.2±1.8
**Apparent growth rate, μ**_app_ **(day^−1^)**	0.14±0.03(R^2^ = 0.99)	0.15±0.07(R^2^ = 0.99)	0.16±0.02(R^2^ = 0.94)
**CRYOPRESERVATION**			
**% cell survival:**			
**Immediately after thawing**	55.7±4.6%	103.7±8.8%	-
**1 day after thawing**	24.9±2.8%	71.0±5.0%	-

Aggregates collected after microcapsule dissolution maintained their integrity and high cell viability (not shown), thus ensuring efficient cell characterization. The results show that hESCs expanded as encapsulated 3D aggregates retained their undifferentiated phenotype during 2 weeks of culture in spinner vessels, as evaluated by immunofluorescence microscopy and flow cytometry (Figure 2E–G). By day 7, the percentages of SSEA-4 and TRA-1-60 positive cells were high (94.6% and 89.2%, respectively), indicating that most cells kept an undifferentiated character (Figure 2E–G). Additionally, for all culture time points the percentages of SSEA-1 positive cells were always below 10% (Figure 2E). At day 18, a significant decrease in SSEA-4 and TRA-1-60 positive cells was observed (Figure 2E); the presence of EB-like structures (aggregates with irregular shape and cystic cavities) at this time (data not shown), suggests that hESCs had started to differentiate.

After alginate dissolution, microencapsulated hESC aggregates expanded in the bioreactor were able to form undifferentiated colonies on top of a monolayer of inactivated hFF (Figure 2H). Moreover, these cells differentiated spontaneously *in vitro*, via EB formation, into cells from the three germ layers (Figure 2I), confirming that they maintained their pluripotent potential.

### Expansion of encapsulated hESC immobilized on microcarriers in stirred tank bioreactors

For the third strategy, hESCs were immobilized on Matrigel-coated Cytodex 3 microcarriers (3 g/L) [7] and encapsulated in alginate. First, the microencapsulation step was tested at different culture time points: 8 h (day 0), and days 1, 3 and 6. Day 6 was selected since it allowed a higher percentage of microcarriers and microcapsule colonization (data not shown). Preliminary experiments also demonstrated that the addition of empty supports (1 g/L) on cell-microcarrier cultures (cells immobilized on microcarriers, 2 g/L, yielding a final concentration of 3 g/L) immediately before microencapsulation, enhanced colonization. Indeed, the addition of empty and freshly coated supports promoted cell migration and further proliferation inside the capsule, ultimately increasing the number of populated microcapsules and cell yields (data not shown).

**Figure 3:**
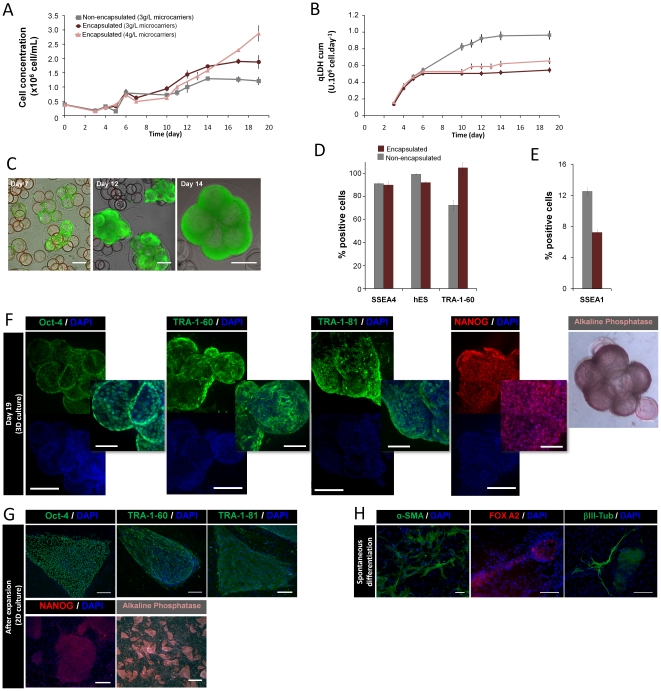
Effect of alginate microencapsulation on the expansion of hESCs immobilized on microcarriers. hESCs were immobilized on Matrigel-coated microcarriers (2 g/L) and encapsulated at day 6. Before microencapsulation empty coated microcarriers (1 g/L and 2 g/L) were added. Non-encapsulated (grey) and encapsulated hESCs using 3 g/L (purple) and 4 g/L (pink) of microcarriers were cultured in spinner vessels. (A) Growth curve expressed in terms of cell number per volume of medium. Error bars denote SD of 3 measurements. (B) Cumulative values of specific rates of LDH release during culture time. Error bars denote SD of 3 measurements. (C) Phase contrast and fluorescence images of encapsulated hESC cultures (on 4 g/L microcarriers) at days 7, 12 and 14. Viability analysis of cultures stained with fluoresceine diacetate (FDA-live cells, green) and propidium iodide (PI- dead cells, red). Scale bar: 200 µm. (D–H) Characterization of encapsulated hESCs immobilized on microcarriers expanded in spinner vessels: (D) Flow cytometry analysis of non-encapsulated (grey bars) and encapsulated (purple bars) hESCs immobilized on microcarriers at the end of the expansion process - percentage of SSEA-4, TRA-1-60 and hES-CellectTM (hES) and (E) SSEA-1 positive cells in relation to the 2D control culture; error bars represent SD of 2 measurements. (F) Confocal images of Oct-4, TRA-1-60, TRA-1-81 and NANOG labeling and phase contrast pictures of alkaline phosphatase (AP) activity at day 19 of encapsulated 3D culture. Nuclei were labeled with DAPI (blue). Scale bar: 200 µm, merge and phase contrast images 100 µm. (G) Immunofluorescence images of Oct-4, TRA-1-60, TRA-1-81 and NANOG labeling after expansion (2D culture). Nuclei were labeled with DAPI (blue). Scale bars: 200 µm and 1 mm for immunofluorescence and phase contrast images, respectively. (H) In vitro pluripotency analysis. Microcapsules were dissolved and hESCs were detached from the microcarriers and transferred to a monolayer of inactivated hFF. At confluence, colonies were dissociated and hESCs were able to form embryoid bodies (EBs) in non-adherent conditions and differentiated into cells from all three germ layers. Fluorescence images of differentiated cultures labeled for α–SMA (α smooth muscle actin, mesoderm), FOX-A2 (Forkheadbox A2, endoderm) and βIII-Tub (β tubulin type III, ectoderm). Nuclei were stained with DAPI (blue). Scale bar: 100 µm.

Encapsulated hESCs immobilized on microcarriers were cultured for 19 days in spinner vessels (Figure 1). The results show that the microencapsulation of cell-microcarriers in alginate markedly enhanced cell viability and expansion when compared to non-encapsulated cultures (Table 1, Figure 3A, B). By day 19, the fold increase in cell concentration was higher in encapsulated (10.7±0.8) than in non-encapsulated (7.7±0.2) cultures, which supports the hypothesis that alginate microcapsules protect the cells from hydrodynamic shear stress, enhancing cell migration and further proliferation on microcarriers. Moreover, no differences were observed in the apparent growth rates (Table 1), indicating that the alginate matrix did not compromise the hESCs proliferation potential.

To further improve cell expansion yields, we increased the concentration of microcarriers: 2 g/L of empty supports were added before microencapsulation, yielding a final concentration of 4 g/L. The increase in available cell growth surface area contributed to augmenting the final cell concentration (2.9×10^6^ cell/mL corresponding to a 19.2±1.8 ratio of expansion, Table 1). Within microcapsules, cells migrated and colonized most of the microcarriers, with total viable cells increasing overtime (Figure 3C). It is important to point out that, using these conditions, the exponential growth phase was prolonged until day 19 (Figure 3A). The culture was aborted at this time because cell overgrowth was observed in some microcapsules (data not shown).

After expansion as encapsulated cell-microcarrier aggregates, hESCs retained their undifferentiated phenotype and pluripotency markers (Figure 3D–F). When compared to non-encapsulated cultures, results were very similar with the exception of TRA-1-60 where higher levels of positive cells were registered in the encapsulated cultures (Figure 3D). The percentage of SSEA-1 positive cells was higher in non-encapsulated (13.0±0.4%) than in encapsulated cultures (7.8±0.3%) (Figure 3E), indicating that, at the end of the expansion process, more cells in an early differentiated state were presented in the former case.

Encapsulated cells maintained the capacity to form undifferentiated colonies in 2D standard monolayer systems (Figure 3G) and presented *in vitro* pluripotency; cells were able to form EBs and spontaneously differentiate into cells from the three embryonic germ layers (Figure 3H).

### Cryopreservation of hESCs using 3D microencapsulated culture strategies

Since hESCs can be successfully expanded in microcapsules as cell aggregates or immobilized on microcarrier surfaces, we evaluated the possibility of cryopreserving these 3D structures and investigated whether microencapsulation in alginate would improve cell viability and survival ratios. Cells were harvested from the bioreactor cultures at specific culture time points (day 13 and 14 for hESCs-microcarriers and aggregate cultures, respectively) (Figure 1) and cryopreserved using the slow rate freezing protocol.

**Figure 4:**
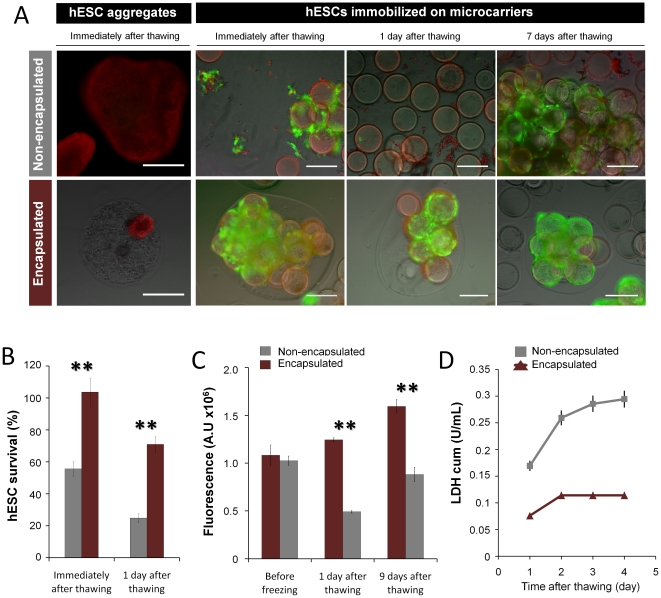
Post-thawing survival of non-encapsulated and encapsulated hESCs. Non-encapsulated and encapsulated hESCs were cryopreserved as aggregates or immobilized on microcarrier using a slow freeze rate method. (**A**) Phase contrast and fluorescence images of cryopreserved hESC immediately, 1, 3 and 7 days after thawing. Viability analysis of hESCs stained with fluoresceinediacetate (FDA-live cells, green) and propidium iodide (PI- dead cells, red). Scale bar: 200 µm. (**B–D**) Post-thawing characterization of non-encapsulated (grey) and encapsulated (purple) hESCs immobilized on microcarriers. (**B**) Percentage of cell survival immediately and one day after thawing. Error bars denote SD of 3 measurements. (**C**) Metabolic activity measured by alamarBlue test before cryopreservation and 1 and 9 days after thawing. Error bars denote SD of 4 measurements. ** indicates significant difference (P<0.05) in metabolic activity by one-way ANOVA. (**D**) Cumulative values of specific rates of LDH release of cryopreserved hESCs after thawing. Error bars denote SD of 3 measurements.

Results showed that in aggregate culture, alginate microencapsulation did not prevent cell death immediately after thawing (Figure 4A). On the contrary, microencapsulated hESCs immobilized on microcarriers presented high cell viabilities and cell recoveries post-thawing (Figure 4A). This strategy was very efficient for the cryopreservation of hESCs as compared to their non-encapsulated culture counterpart; immediately and one day after thawing, the percentage of cell survival was significantly higher in encapsulated (day 0 = 103.7±8.8%, day 1 = 71.0±5.0%) than in non-encapsulated cells (day 0 = 55.7±4.6%, day 1 = 24.9±2.8%) (P<0.05) (Figure 4B, Table 1). Although some cell death occurred in the first days post-thawing, microencapsulated hESCs immobilized on microcarriers more quickly recovered their proliferative and metabolic activity (Figure 4C). In non-encapsulated cultures, cells were prone to detach from the microcarriers after thawing resulting in pronounced levels of cell death (Figure 4A); additionally, cells did not re-establish their metabolic activity and the values of LDH were higher than in encapsulated cultures at all time points (Figure 4C–D).

**Figure 5:**
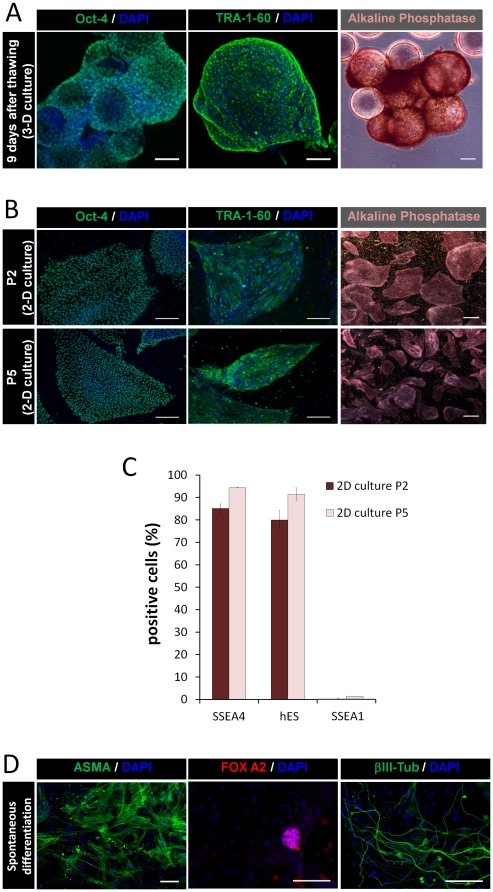
Post-thawing characterization of encapsulated hESCs immobilized on microcarriers. Phenotype analysis of encapsulated hESC immobilized on Matrigel coated Cytodex3 microcarriers (**A**) 9 days post-thawing (P0) and (**B**) after 2 and 5 cell passages in 2D culture systems (P2 and P5, respectively); confocal images of Oct-4, and TRA-1-60 labeling and phase contrast pictures of alkaline phosphatase (AP) activity. Nuclei were labeled with DAPI (blue). Scale bars: (**A**) 100 µm and (**B**) 200 µm for immunofluorescence images; (**A**, **B**) 1 mm for phase contrast images. (**C**) Flow cytometry analysis; percentage of SSEA-4, hES-Cellect™ (hES) and SSEA-1 positive cells after 2 and 5 cell passages post-thawing (P2 and P5, respectively). Error bars represent SD of 2 measurements. (**D**) *In vitro* pluripotency analysis. Microcapsules were dissolved and hESCs were detached from the microcarriers and transferred to a monolayer of inactivated hFF. At confluence, colonies were dissociated and hESCs were able to form embryoid bodies (EBs) in non-adherent conditions and differentiated into cells from all three germ layers. Fluorescence images of differentiated cultures labeled for α–SMA (α smooth muscle actin, mesoderm), FOX-A2 (Forkheadbox A2, endoderm) and βIII-Tub (β tubulin type III, ectoderm). Nuclei were stained with DAPI (blue). Scale bars: 100 µm.

To investigate whether microencapsulated hESCs immobilized on microcarriers maintained their pluripotent characteristics after cryopreservation, cells were characterized 9 days post-thawing and during 5 additional passages on top of inactivated hFF monolayers. The results confirmed that hESCs maintained their undifferentiated phenotype, pluripotency markers (Figure 5A–C) and the ability to differentiate *in vitro* into cells from the three germ layers (Figure 5D).

## Discussion

Efficient culture strategies are urgently needed to accelerate the transition of hESCs to clinical and industrial applications. This study intended to develop an integrated bioprocess for the expansion and cryopreservation of pluripotent hESCs; our approach consisted of designing 3D culture strategies using cell microencapsulation in alginate. Results show that the combination of cell microencapsulation and microcarrier technology is an optimum process for the scalable production and storage of high-quality pluripotent hESCs.

Microencapsulation in alginate proved valuable for improving hESC expansion in stirred tank bioreactors, since it ensures a shear stress free microenvironment and avoids excessive clustering of microcarriers or aggregates in culture. This strategy is extremely attractive for use in large-scale bioprocesses, enabling tighter control of the culture and higher cell expansion yields than non-encapsulated cultures.

Our results show that the microencapsulation of hESCs immobilized on microcarriers is also very efficient strategy for the long-term culture of undifferentiated cells, overcoming the limitations of both single cells and aggregate cultures. Cell-cell and cell-matrix interactions significantly affect stem cell fate decisions (apoptosis, self-renewal, differentiation) (reviewed in [3], [33]). Our results are in agreement, showing that these interactions lead to improved stem cell bioprocesses. In fact, hESCs drastically lose their viability when encapsulated as single cells, even after treatment with Y-27632, a selective ROCK inhibitor known to prevent apoptosis of hESCs after single cell enzymatic dissociation [31], [34]. When encapsulated as aggregates, hESCs tend to spontaneously differentiate after 2 weeks of culture; this might be explained by the observed increase in aggregate size (>250 µm), which may limit the diffusion of growth factors and gas substrates within aggregates, thereby inducing the formation of EB-like structures and reducing cell proliferation capacity. In a previous study, Siti-Ismai *et al.* reported the long-term feeder-free cultivation of hESC aggregates in large (approximately 1 mm) calcium alginate capsules, confirming that cells retained their undifferentiated state and pluripotent characteristics for up to 260 days [12]. This difference in cell behavior may reflect the distinct hESC line and/or culture conditions (alginate matrix, culture medium) used. Nevertheless, the culture of microencapsulated hESC aggregates could be adopted for the production of human stem cell derivatives, by inducing directed differentiation at the second week of culture (when stem cell population is still pluripotent) and bypassing the EB formation step in a controlled manner. There are several studies reporting the use of this strategy to differentiate mouse and/or human ESCs into pancreatic insulin-producing cells [28], hepatocytes [26] definitive endoderm [31], cardiomyocytes [25] and osteoblasts [24]. High expectations are raised by these culture strategies to potentiate the use of hESCs in cell therapy and tissue engineering applications (reviewed in [35]).

Another advantage of microcarrier technology in stem cell expansion processes is the flexibility with which the area available for cell growth can be adjusted, further facilitating the process scale-up. From the clinical and industrial perspectives, this scalability would have tremendous impact in reducing the costs of cell manufacturing by cutting the media, growth factors and other expensive supplements needed for stem cell cultivation [36]. Increasing the concentration of microcarriers permitted us to reach up to 3×10^6^ cell/mL, corresponding to a 15-fold increase in final cell yields when compared to standard 2D protocols [7] (Table 2). Although performed in small lab scale spinner vessels, the strategies developed hereby can be easily up-scaled to environmentally controlled stirred tank bioreactors where scalability, automation and accurate control of culture environment are guaranteed; our group has recently shown that the expansion of pluripotent hESCs can be further improved in stirred tank bioreactors with controlled pO_2_ and continuous perfusion [7].

**Table 2:** Characteristics and bioprocess yields of the 3D strategy and the standard 2D protocols.

	2D Strategy(colonies culture)	3D Strategy(microencapsulated cells-microcarriers)
Culture system	Adherent, Static(well plates, Petri dishes)	Suspension, Stirred(Stirred tank bioreactors, spinner vessels)
Ease of monitorization	Yes	Yes
Ease of handling	Yes	Yes
Ease of scale-up	No	Yes
Time- and space- consuming	Yes	No
Reproducibility	Low	High
Mimicry stem cells' native microenvironment	No	Yes
Affordability	Yes	costs associated to encapsulation equipment/process and material (microcarriers, hydrogels)
Expansion ratios	11 ^[7]^	20
Maximum Cell Concentration (×10^6^ cell/mL)	0.2 ^[7]^	3
Cell survival after cryopreservation (slow freezing rate)	<20% ^[5]^	>70%

This study further established that microencapsulation of hESCs immobilized on microcarriers is an efficient process for the cryopreservation of hESCs, since it allows for the recovery of undifferentiated hESCs with high viabilities and the maintenance of their pluripotent characteristics over several passages under standard culture conditions, enabling their use for further applications. The presence of components of the extracellular matrix on microcarrier cultures (e.g. collagen, laminin) may contribute to enhance cell survival during freezing and thawing [37], [38], by reducing post-thaw apoptosis [5], [37]. In contrast, microencapsulated aggregates showed high cell death immediately after thawing. The limitations in heat and mass (water and cryoprotectant) transfer within the aggregates may result in different cryoprotection gradients, possibly leading to cryodamage [30], [39]. More fundamental studies on the physico-chemical and biophysical phenomena occurring during freezing/thawing of microencapsulated hESC aggregates should allow for further improvement of this process.

It is important to highlight that the cryopreservation of hESCs immobilized on microcarriers has already been reported by Nie *et al* [10]. The key advantage of our strategy is that higher cell recovery yields can be achieved without the use of feeder cells. The alginate microcapsule allows further improvement of post-thaw cell viability, enhancing up to a 3-fold boost in cell survival compared to non-encapsulated cultures. Although the underlying mechanisms are still unclear, several studies indicate that maintaining cell-cell/matrix contacts improves hESC recovery following cryopreservation [37], [40]. Cell entrapment within alginate microcapsules may help protect hESCs from the adverse effects of cryopreservation, not only by preventing the disruption of cell-cell and cell-matrix contacts [21], [30] but also by decreasing exposure to cryoprotectants and preventing the damage caused by intracellular ice formation and propagation (via gap junctions) [41].

This is the first time that the successful expansion and cryopreservation of pluripotent hESCs on microcarriers inside alginate microcapsules have been reported. Moreover, an integrated bioprocess for the efficient production, banking and distribution of hESCs in a scalable and straightforward manner is now possible. The main limitations of this 3D strategy, when compared to the standard 2D protocols, are the costs associated with the encapsulation equipment/process and material (microcarriers and alginate) (Table 2). But its inherent scalability and reproducibility and the high bioprocess yields associated with the 3D approach (Table 2) should more than compensate. Hopefully, the integrated strategy developed herein will potentiate hESCs to achieve a wider range of applications. As hESCs can be harvested from the microcapsules with high viability and pluripotency, they could have immediate use for *in vitro* applications demanding high numbers of cells, e.g. in high-throughput screening of pharmaceutical compounds. However, from a clinical perspective, further improvements are still required including the adaptation to defined xeno-free culture conditions and the integration of a directed differentiation step. The presence of microcarriers within the microcapsules is still a concern, requiring an additional step to release cells from the microcapsules and separate them from the microcarriers before cell transplantation; alternatively, a biodegradable, clinically approved microcarrier could be used. Indeed, gelatin and pharmacologically active microcarriers (PAMs) have been used successfully in adult cell therapy for brain neuronal damage and cartilage engineering (reviewed in [42]). Although the type of alginate used in this study has never been tested in clinical studies, it is manufactured in compliance with current GMP and presents low levels of endotoxins (≤100 EU/g), conditions that may boost its use in transplantation experiments.

In conclusion, the experiments herein described demonstrate that cell microencapsulation in alginate is a powerful tool for integrating expansion and cryopreservation of pluripotent hESCs. Moreover, the combination of cell microencapsulation with microcarrier technology promotes cellular interactions that are essential for improved production and storage of hESCs without compromising their viability, self-renewal and pluripotency. This 3D culture strategy represents an important step for enlarging the range of hESC applications for regenerative medicine, tissue engineering and *in vitro* toxicology. Future studies will incorporate a differentiation step so as to develop a fully integrated bioprocess for the expansion, differentiation and storage of clinically relevant hESC derivatives.

## Supporting Information

Figure S1 **Microencapsulation of hESCs as single cells in alginate.** Phase contrast and fluorescence images of hESC encapsulated at 2×10_6_ cell/mL alginate, by day 1 (A) and day 7 (B) of culture. Viability analysis of cultures stained with fluoresceine diacetate (FDA-live cells, green) and propidium iodide (PI- dead cells, red). Scale bar: 200 µm.
(TIF)
