# Supplementary figures and images for: Microencapsulation Technology: A Powerful Tool for Integrating Expansion and Cryopreservation of Human Embryonic Stem Cells
Source: PLoS One. 2011 Aug 5;6(8):e23212. doi: 10.1371/journal.pone.0023212 (PMC3151290; doi:10.1371/journal.pone.0023212)

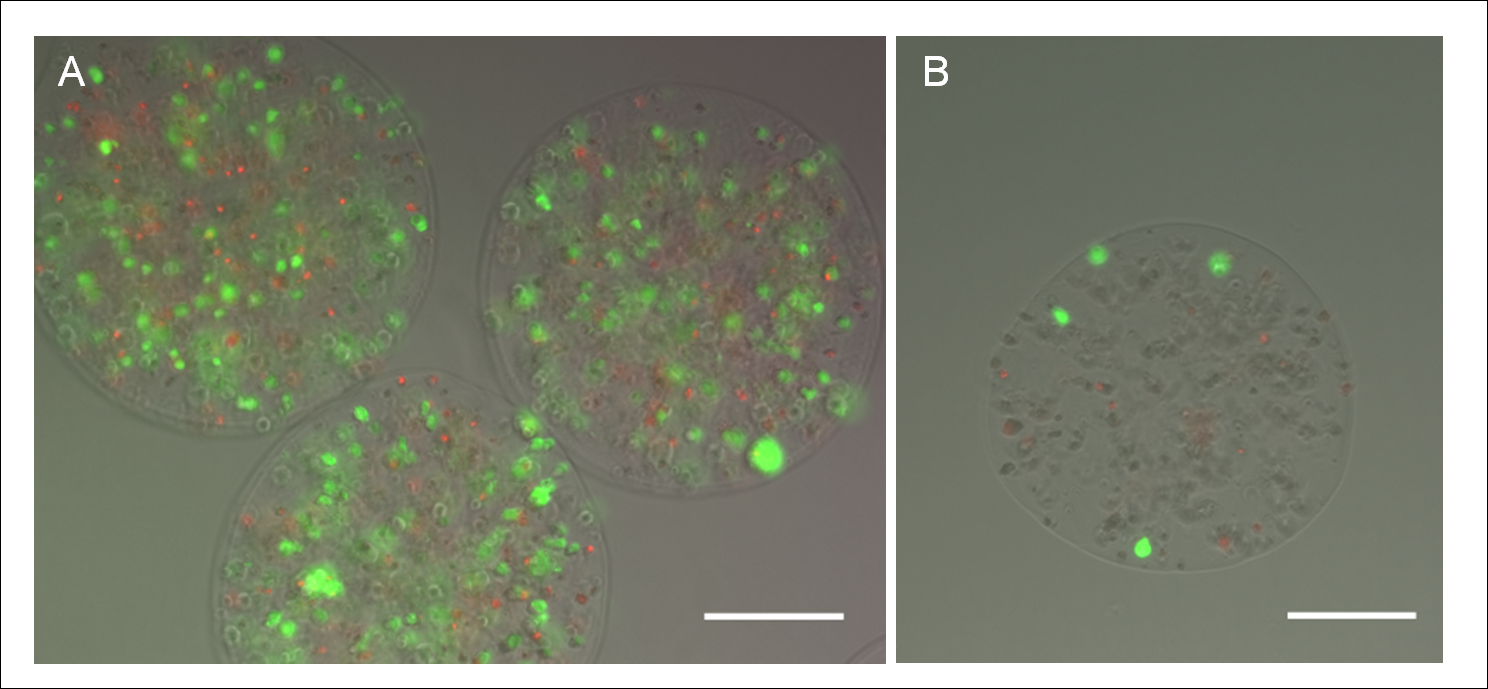

Supplement: Figure S1 — Microencapsulation of hESCs as single cells in alginate. Phase contrast and fluorescence images of hESC encapsulated at 2×10^6^ cell/mL alginate, by day 1 (A) and day 7 (B) of culture. Viability analysis of cultures stained with fluoresceine diacetate (FDA-live cells, green) and propidium iodide (PI- dead cells, red). Scale bar: 200 µm. (TIF) [file pone.0023212.s001.tif]
